# Synthesis of end-user research to inform future multipurpose prevention technologies in sub-Saharan Africa: a scoping review

**DOI:** 10.3389/frph.2023.1156864

**Published:** 2023-05-31

**Authors:** Nivedita L. Bhushan, Kathleen Ridgeway, Ellen H. Luecke, Thesla Palanee-Phillips, Elizabeth T. Montgomery, Alexandra M. Minnis

**Affiliations:** ^1^ RTI International, Research Triangle Park, NC, United States; ^2^ Johns Hopkins Bloomberg School of Public Health, Baltimore, MD, United States; ^3^ WITS-RHI, University of the Witwatersrand, Johannesburg, Gauteng, South Africa

**Keywords:** multipurpose prevention technologies, HIV, contraception, pregnancy, end-users, review

## Abstract

**Introduction:**

Women in sub-Saharan Africa (SSA) experience disproportionately high rates of HIV infection and unintended pregnancy compared to their age-matched counterparts in other regions of the world. Multipurpose prevention technologies (MPTs) that offer protection against HIV and unintended pregnancy in a single product stand to address these dual sexual and reproductive health needs simultaneously. The aim of this scoping review is to identify factors that are important for optimizing the likelihood of MPT adoption by end users in SSA.

**Methods:**

Study inclusion criteria included MPT research (HIV and pregnancy prevention dual indication) published or presented in English from 2000 to 2022 and conducted in SSA amongst end-users (women aged 15–44), male partners, health care providers, and community stakeholders. References were identified by searching peer reviewed literature, grey literature, conference presentations (2015–2022), grant databases, and outreach to MPT subject matter experts. Of 115 references identified, 37 references met inclusion criteria and were extracted for analysis. A narrative synthesis approach was used to summarize findings within and across MPT products.

**Results:**

Studies were identified from six countries in SSA and a substantial proportion included a South African (*n* = 27) and/or Kenyan (*n* = 16) study site. Most studies utilized a qualitative study design (*n* = 22) and evaluated MPT acceptability and preferences by presenting hypothetical products through images or a list of product attributes (*n* = 21). The vaginal ring (*n* = 20), oral tablet (*n* = 20), and injection (*n* = 15) were examined most frequently. Across studies, there was high acceptability and demand for an HIV and pregnancy prevention MPT. End users valued choice in prevention product type as well as discreetness and long-acting options. Provider counseling and community sensitization were reported as essential for future introduction of novel MPT delivery forms.

**Conclusion:**

Recognizing the heterogeneity of women's preferences and changing reproductive and sexual health needs over the life course, choice is important in the delivery of pregnancy and HIV prevention products as well as amongst MPT products with distinct product profiles. End user research with active MPTs, vs. hypothetical or placebo MPTs, is necessary to advance understanding of end-user preferences and acceptability of future products.

## 1. Introduction

In sub-Saharan Africa (SSA), adolescent girls and young women (AGYW) ages 15–24 account for nearly 32% of all new HIV infections, and 40%–65% report an unintended pregnancy before the age of 25. This sexual and reproductive health burden among AGYW in the SSA region is disproportionally high compared to their age-matched counterparts in other regions of the world (1, 2) and persists despite significant progress in HIV and unintended pregnancy prevention over the last decade, including increased availability of and access to contraceptive options, opt-out HIV testing and counseling, voluntary medical male circumcision, treatment for HIV-positive individuals, and pre-exposure prophylaxis (PrEP) available in oral tablet, and, most recently, vaginal ring and injectable formulations (3).

Multipurpose prevention technologies (MPTs) that offer protection against HIV and unintended pregnancy in a single product stand to address these dual sexual and reproductive health needs simultaneously (4, 5). MPTs have the potential for increased acceptability and use relative to single-indication products for numerous reasons (6–8). First, improved access, consistent use, and health system efficiencies could be achieved through offering an integrated product that requires fewer clinic visits and reduces provider burden. Second, reductions in stigma related to HIV prevention product use could be achieved by developing discreet MPT products and integrating MPTs into family planning delivery systems and messaging. Third, increased uptake could be achieved by ease of MPT use and expanded choice in the available method mix (6, 7, 9). Male and female condoms, however, are the only approved MPTs available.

The existing MPT research and development pipeline includes a diverse range of delivery forms, mechanisms of action, and indications (10–12). Vaginal rings, which contain both antiretroviral and contraceptive agents, offer 1- or 3-month continuous use and constitute the delivery form with the greatest number of products in development, including both nonhormonal and hormonal rings (11, 13). The co-formulated dual prevention pill (DPP) is anticipated to be the first MPT to move to market since female and male condoms; the pharmacokinetic profile of a co-formulated DPP is being assessed in a bioequivalence trial. Acceptability of an over-encapsulated DPP is also being evaluated through two studies in Zimbabwe and South Africa (14, 15). Vaginally delivered products comprise a core focus of the future MPT pipeline, with both on-demand forms used prior to intercourse (such as fast-dissolving inserts) and, more recently, longer-acting formulations (such as monthly films) in preclinical development and planned early clinical trials. Other long-acting MPT delivery forms, such as an implant and a microneedle applicator patch, are also in preclinical development (12).

While active MPT products are largely in the design and research phase, there have been studies conducted to explore MPT acceptability by presenting women with hypothetical MPT products through images and product attribute lists or providing women with placebo MPT products for use. This review synthesizes what is known about end user preferences for MPTs for HIV and pregnancy prevention in the existing literature and identifies gaps in the evidence base. This information is essential to inform the development of new MPTs for prevention of unintended pregnancy and HIV. The overarching goal of this scoping review is to identify what product attribute factors and social factors are important for optimizing the likelihood of MPT adoption and use by end users. Thus, we examine the existing evidence on MPT preferences and acceptability amongst end users and how they are viewed and influenced by male partners, health care providers, and other community stakeholders in SSA.

## 2. Methods

### 2.1. Scoping review

We conducted a scoping review, which enables researchers to map the current state of research and identify gaps in knowledge. Unlike systematic reviews, scoping reviews are intended to explore multiple research questions without restrictions on a particular study design and readily allows for inclusion of conference abstracts and unpublished reports (16). Scoping reviews are often precursors to systematic reviews and meta-analyses because they can be used to confirm the relevance of inclusion criteria and research questions for future research and synthesis efforts.

### 2.2. Search terms and inclusion criteria

**Figure 1:**
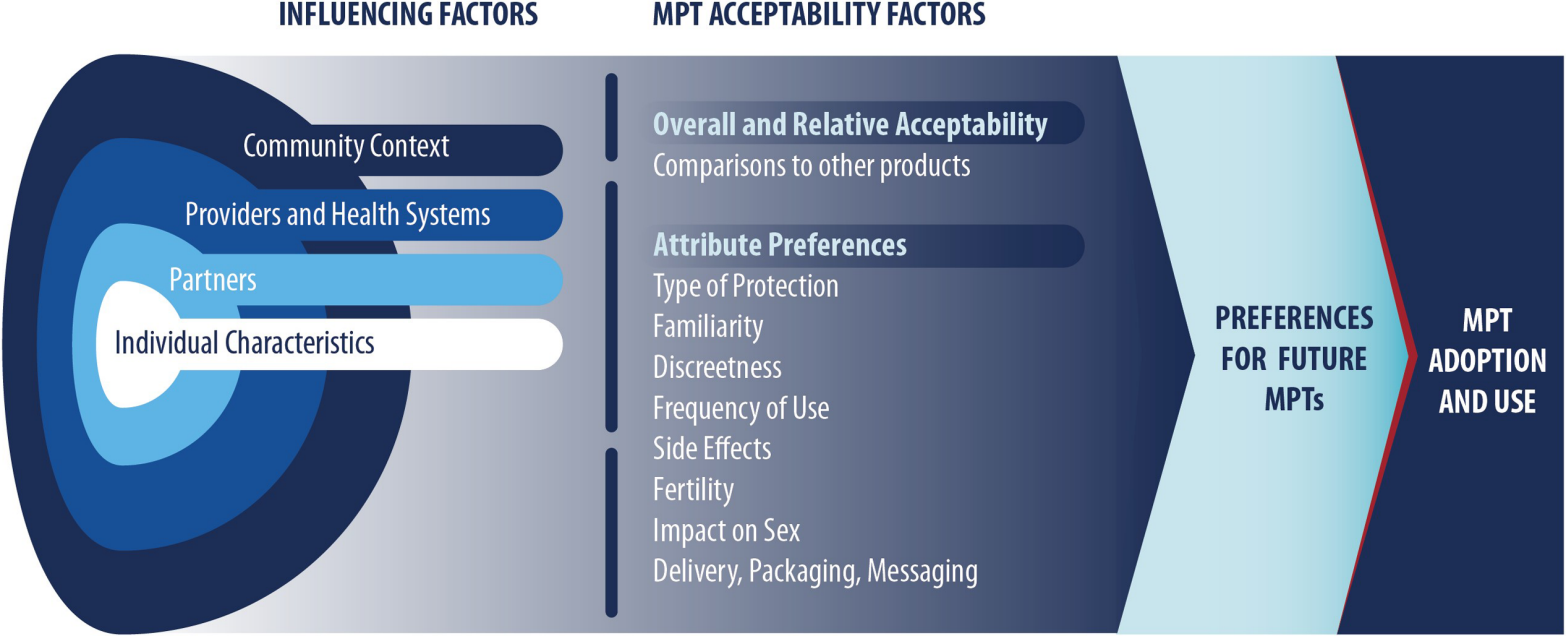
Conceptual model.

The conceptual model used for the present review (Figure 1) informed our selection of search terms and synthesis of resulting articles. The conceptual model was refined drawing on two existing frameworks [Mensch et al. (17); Friedland et al. (14)] that were developed to be HIV PrEP or MPT product specific. The Mensch et.al., framework suggests that influencing factors and acceptability factors impact product preference and adherence (17), which in this review applies to use of future products. Influencing factors are based on the socio-ecological model, whereas acceptability factors are based on product-specific attributes and perceptions. The Friedland et al. framework suggests that provider factors and product factors inform an individual's HIV and pregnancy prevention choices and ultimately their intention to use future MPTs (14).

Search terms were also informed by our inclusion criteria. Study inclusion criteria included research published or presented between January 1, 2000 and November 30, 2022, in English and with a geographic location in one or more sub-Saharan African location. We included original research regardless of study design, research encompassing all delivery forms in peer-reviewed literature or the MPT development pipeline, and specifically focused on MPTs designed to combine HIV and pregnancy prevention. We excluded research that reported on condoms only as an MPT and peer-reviewed publications that reported modeling studies, reviews, commentaries, and editorials. A list of search terms is included in Supplementary Table S1.

### 2.3. Reference identification

The study team used multiple search modalities to identify relevant references. To comprehensively search the peer-reviewed literature, the study team worked with a research librarian to develop a structured search strategy for articles indexed on PubMed, Embase, and Web of Science. The study team then conducted extensive hand-searching to identify relevant conference abstracts, grey literature reports, and manuscripts under review not available in the above databases. Hand-searching included a comprehensive search of MPT and HIV prevention websites (i.e., AVAC, IMPT, PrEP Watch), a search of HIV prevention and family planning conferences [i.e., International AIDS Conference (AIDS), IAS Conference on HIV Science (IAS), HIV Research for Prevention Conference (HIVR4P), Conference on Retroviruses and Opportunistic Infections (CROI), Population Association of America Annual Meeting (PAA), International Conference on Family Planning (ICFP)] held between 2015 and 2022, and a review of the reference lists of the included articles. To orient the scoping review to MPT products in the development pipeline and ongoing MPT-related research, we conducted a search of NIH RePORTER and Grants.gov and reached out to investigators with current funded research and known MPT subject matter experts regarding their ongoing and future work.

### 2.4. Synthesis approach

All references identified in the search process were uploaded to Covidence, an online review software. Two study team members independently reviewed each reference by title and abstract, and then by full text, applying specified inclusion criteria. Structured forms were used to extract information from the resulting set of included references. Team members met to discuss differences when individual determinations did not align to reach consensus at each stage. A narrative synthesis approach was then used to summarize findings within and across products. Narrative synthesis is an appropriate strategy in scoping and other reviews when variability across study designs and outcomes assessed preclude our ability to use meta-analytic techniques. For this review, we read through all extracted text and identified relevant thematic categories that appeared frequently in extracted text (e.g., familiarity, discreetness) through discussion with one another and consultation with our conceptual framework. After reaching consensus on these themes, we created product-specific summaries that pulled together all end-user data for a specific MPT product type and narratively summarized available data on each theme, noting gaps in the available literature and any studies that stratified results by region or sociodemographic characteristics. Finally, we compared findings across these product-specific summaries and created cross-product syntheses, which draw upon common findings identified across products for the same theme and highlighted distinctions and gaps in the evidence. This process was similar to a qualitative data analysis through coding and memo-writing. The larger research team held meetings to discuss overall emerging themes and to identify gaps in the evidence that warranted further exploration.

## 3. Results

### 3.1. Overview of studies

**Figure 2:**
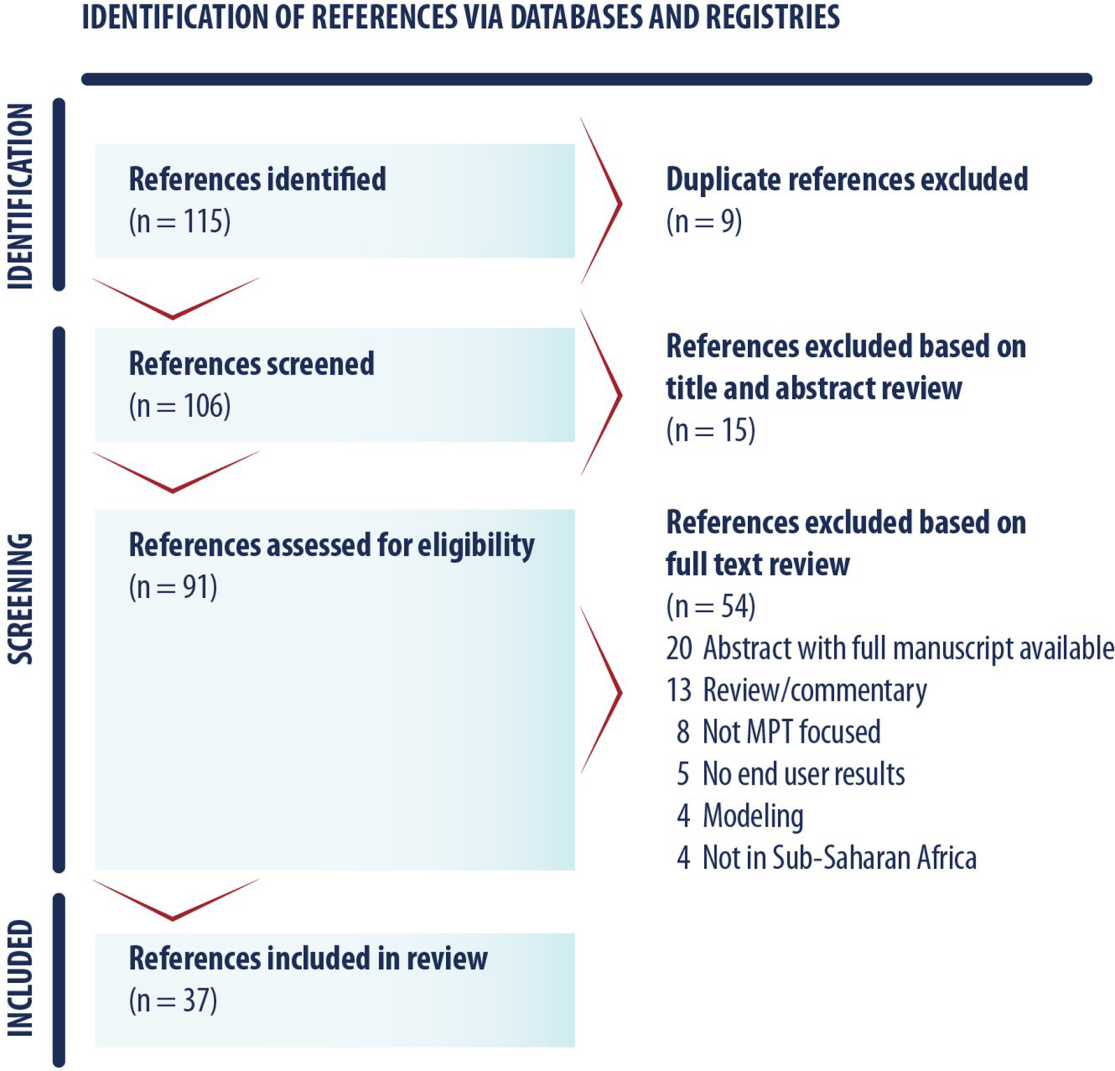
PRISMA diagram.

As shown in Figure 2, the team identified 113 unique references and 37 were included in the review, with reasons for exclusion noted. A summary of key characteristics of included references presented in Table 1 with a full list of references is available in Table 2. Most references came from the peer-reviewed literature (*n* = 21) followed by conference findings (*n* = 10), and grey literature (*n* = 6). The most frequently used study design was qualitative (*n* = 22), followed by a variety of quantitative approaches (i.e., discrete choice experiment (DCE; *n* = 6) and randomized cross-over (*n* = 3), mixed methods (*n* = 3), and human-centered design workshops (*n* = 2). Study sites spanned six countries in sub-Saharan Africa: South Africa, Kenya, Zimbabwe, Uganda, Nigeria, and Malawi. A substantial proportion of studies included a South African study site (*n* = 27) and/or a Kenyan study site (*n* = 16), reflective of many articles that included data from the Tablets, Ring, and Injectables as Options (TRIO) study, which examined acceptability of placebo versions of these three delivery forms for an MPT indication (*n* = 11) (53).

**Table 1:** Reference characteristics (*N* = 37).

	*n*	%
Publication Type		
Peer-Review Article	21	57%
Conference Findings	10	27%
Grey Literature	6	16%
Country^a^		
South Africa	27	73%
Kenya	16	43%
Zimbabwe	12	32%
Uganda	9	24%
Nigeria	1	3%
Malawi	1	3%
Years		
2013–2017	6	16%
2018	7	19%
2019	5	14%
2020	2	5%
2021	5	14%
2022	11	30%
Study Design		
Qualitative	22	59%
Discrete Choice Experiment	6	16%
Randomized Cross-Over	3	8%
Mixed Methods	4	11%
HCD Workshops	2	5%
Other Influential Populations^a^		
Providers	7	19%
Male Partners and Men	12	32%
Community Stakeholders	5	14%
Number of Participants		
Not reported	4	11%
<100	15	41%
100–500	10	27%
>500	8	22%
Hypothetical or Actual Products		
Placebo MPT Products	14	38%
Hypothetical Products	21	57%
Active MPT Products	2	5%
Participant Interaction with MPTs^a^		
Used product(s)	11	30%
Did Not Use: Saw Pictures	21	57%
Did Not Use: Touched Products	6	16%
Product Type^a^		
Vaginal Ring	20	54%
Oral Tablet	20	54%
Injectable	15	41%
Vaginal Microbicide Gel	7	19%
Diaphragm	5	14%
Vaginal Film	5	14%
Subcutaneous Implant	5	14%
Vaginal Insert	3	8%
Hypothetical Vaginal MPT	2	5%
Vaginal Fabric^b^	1	3%
Microarray Patch	3	8%
Parent Study		
TRIO	11	30%
QUATRO	2	5%
MTN-045/CUPID	3	8%
Fabric Study	1	3%
HPTN-035 and Duet	1	3%
SCHEILD	2	5%
UPTAKE	1	3%
Kisumu Combined Ring Study	1	3%
Not named	16	43%

^a^ Totals greater than 100% due to category overlap.

^b^ The vaginal fabric is a novel dosage form for intravaginal drug delivery made of drug-eluting nanofibers.

**Table 2:** References reporting MPT acceptability and preferences.

Author, Year	Reference Type	Country	Study/Trial Name	Study Design	Population	Sample Size	Product Type(s)	Product Use
Agot, 2019 (18)	Peer-Reviewed Article	Kenya; South Africa	TRIO	Qualitative Study	Women (age 18–30)	277	Ring; Oral tablet; Injectable	Placebo Products
Agot, 2020 (19)	Peer-Reviewed Article	Kenya; South Africa	TRIO	Qualitative Study	Women (age 18–30)	165	Ring; Oral tablet; Injectable	Placebo Products
AVAC, 2021 (20)	Grey Literature	South Africa; Zimbabwe	None	Human Centered Design Workshops	Women (age 18+)	25	Oral tablet	Hypothetical Products
Barker, 2021 (21)	Conference Findings	South Africa; Zimbabwe	None	Qualitative Study	Adolescent and Adult Women and Men (age 16–40)	Not Reported	Oral tablet	Hypothetical Products
Bayigga, 2018 (22)	Conference Findings	Uganda	DREAM Trial	Qualitative Study	Community Stakeholders	1,076	Ring	Hypothetical Products
Beksinska, 2018 (23)	Peer-Reviewed Article	South Africa	None	Randomized Cross-Over Study	Women (age 18–45)	115	Gel; Diaphragm	Placebo Products
Bhushan, 2022 (24)	Peer-Reviewed Article	Uganda; Zimbabwe	MTN-045/CUPID	Qualitative Study	Couples (Women (age 18–40), Men (age 18+)	78 (39 couples)	Ring; Oral tablet	Hypothetical Products
Bowen, 2017 (25)	Grey Literature	South Africa	None	Qualitative Study	Adolescent Girls, Adolescent Boys, Women, Men (age 16–34)	28	Ring	Hypothetical Products
Browne, 2020 (26)	Peer-Reviewed Article	South Africa; Zimbabwe	QUATRO	Discrete Choice Experiment	Women (age 18–30)	395	Vaginally Delivered MPT	Hypothetical Products
Gachigua, 2022 (27)	Conference Findings	Kenya	None	Qualitative Study	Adolescent Girls and Young Women (age 15–24), Female Sex Workers, male partners of AGYW/FSW, Stakeholders	Not Reported	Microarray Patch	Placebo Products
Gachigua, unpublished (28)	Conference Findings	Kenya	None	Qualitative Study	Adolescent Girls and Young Women (age 15–24), Female Sex Workers, male partners of AGYW/FSW, Stakeholders	Not Reported	Microarray Patch	Placebo Products
Ipsos, 2014 (29)	Grey Literature	Nigeria; South Africa; Uganda	None	Mixed Methods Study	Women (age 15–35), Men (age 18+)	2,165 (Qualitative Sample: 443; Quantitative Sample: 1,722)	Ring; Implant; Injectable; Film	Hypothetical Products
Kilbourne-Brook, 2021 (30)	Conference Findings	South Africa; Uganda	None	Qualitative Study	Women (ages 18–24 years), Female Sex Workers, Heterosexual men, MSM, Stakeholders	Not Reported	Microarray Patch	Hypothetical Products
Laborde, 2018 (31)	Peer-Reviewed Article	South Africa; Uganda; Zimbabwe	Fabric Study	Qualitative Study	Women (age 18–49)	55	Gel; Film; Fabric	Placebo Products
Lunani, 2022 (32)	Conference Findings	Kenya; Uganda	UPTAKE	Qualitative Study	Adolescent Girls and Women (age 15–24)	30	Injectable	Hypothetical Products
Lutnick, 2019 (33)	Peer-Reviewed Article	Kenya; South Africa	TRIO	Qualitative Study	Women (age 18–30)	24	Ring; Oral tablet; Injectable; Implant	Placebo Products
MatCH Research, 2016 (34)	Grey Literature	South Africa	None	Qualitative Study	Women (age 18–49), Men (age 18+)	24	Gel; Diaphragm	Hypothetical Products
McLellan-Lemal, 2022 (35)	Peer-Reviewed Article	Kenya	Kisumu Combined Ring Study	Qualitative Study	Women (age 18–34)	25	Ring	Active Product
Mgodi, 2022 (36)	Conference Findings	Kenya; South Africa; Zimbabwe	None	Human Centered Design Workshops	Not Reported	Not Reported	Oral tablet	Hypothetical Products
Milford, 2014 (37)	Conference Findings	South Africa	None	Qualitative Study	Women and Stakeholders	24	Diaphragm	Hypothetical Products
Minnis, 2018 (38)	Peer-Reviewed Article	Kenya; South Africa	TRIO	Mixed Methods Study	Women (age 18–30)	277	Ring; Oral tablet; Injectable	Placebo Products
Minnis, 2019a (39)	Peer-Reviewed Article	Kenya; South Africa; Zimbabwe	TRIO; QUATRO	Mixed Methods Study	Women (age 18–30)	419	Ring; Oral tablet; Gel; Injectable; Film; Insert	Placebo Products
Minnis, 2019b (41)	Peer-Reviewed Article	Kenya; South Africa	TRIO	Discrete Choice Experiment	Women (age 18–30)	536	Ring; Oral tablet; Injectable	Placebo Products; Hypothetical Products
Minnis, 2021 (6)	Peer-Reviewed Article	Kenya; South Africa	TRIO	Qualitative Study	Women (age 18–30)	88	Ring; Oral tablet; Injectable	Placebo Products
Minnis, 2022 (42)	Peer-Reviewed Article	Uganda; Zimbabwe	MTN-045/CUPID	Discrete Choice Experiment	Couples (Women (age 18–40), Men (age 18+))	800 (400 couples)	Ring; Oral tablet; Film; Inserts	Hypothetical Products
Namukwaya, 2022 (43)	Conference Findings	Uganda	None	Qualitative Study	Adolescent Girls and Adult Women Sex Workers (age 15–45)	15	Oral tablet; Implant; Injectable; Hypothetical Vaginal Product	Hypothetical Products
Nkomo, 2021 (44)	Conference Findings	South Africa; Zimbabwe	SCHEILD Study	Qualitative Study	Women (age 18–30)	110	Implant	Hypothetical Products
Nkomo, Under Review (45)	Grey Literature	South Africa; Zimbabwe	SCHIELD	Qualitative Study	Women (age 18–30)	110	Implant	Hypothetical Products
Quaife, 2018 (46)	Peer-Reviewed Article	South Africa	None	Discrete Choice Experiment	Adolescent Girls (age 16–17), Women and Men (age 18–49), Female Sex Workers	661	Ring; Oral tablet; Gel; Injectable; Diaphragm	Hypothetical Products
Routes2Results, 2017 (47)	Grey Literature	South Africa	None	Mixed Methods Study	Women (age 18–21)	1,457 (Qualitive Sample: 216, Quantitative Sample: 1,241)	Ring; Oral tablet	Hypothetical Products
Shapley-Quinn, 2019 (40)	Peer-Reviewed Article	Kenya; South Africa	TRIO	Qualitative Study	Women (age 18–30)	88	Ring; Oral tablet; Injectable	Placebo Products
Stoner, 2022 (48)	Peer-Reviewed Article	Uganda; Zimbabwe	MTN-045/CUPID	Discrete Choice Experiment	Couples (Women (age 18–40), Men (age 18+))	790 (395 couples)	Ring; Oral tablet; Film; Insert	Hypothetical Products
Terris-Prestholt, 2013 (49)	Peer-Reviewed Article	South Africa	None	Discrete Choice Experiment	Women (age 18–45)	1,017	Microbicide	Hypothetical Products
Wagner, 2022 (51)	Peer-Reviewed Article	Kenya; South Africa	TRIO	Qualitative Study	Women (age 18–30)	127	Ring; Oral tablet; Injectable	Placebo Products
Weinrib, 2018 (50)	Peer-Reviewed Article	Kenya; South Africa	TRIO	Randomized Cross-Over Study	Women (age 18–30)	277	Ring; Oral tablet; Injectable	Placebo Products
Woodsong, 2014 (52)	Peer-Reviewed Article	Zimbabwe; Malawi	HPTN 035A, Duet Acceptability Study	Qualitative Study	Women (age 18+)	231	Gel; Diaphragm	Active Product
van der Straten, 2018 (53)	Peer-Reviewed Article	Kenya; South Africa	TRIO	Randomized Cross-Over Study	Women (age 18–30)	277	Ring; Oral tablet; Injectable	Placebo Products

Given the diversity in type of study design, sample sizes ranged from 15 participants to 2,165 participants; however, most studies included fewer than 200 participants. Additionally, most references included end users aged 15–24. Although AGYW perspectives therefore predominate, the literature also included perspectives from health care providers (*n* = 7), men and male partners (*n* = 12), and community stakeholders (*n* = 5). Most references evaluated MPT acceptability and preferences by presenting potential future products (*n* = 21) where participant interaction with MPT candidates was limited to seeing images of candidate products in the pipeline and/or seeing a list of potential product attributes (*n* = 20). The vaginal ring (*n* = 20), oral tablet (*n* = 20), and injectable (*n* = 15) were most frequently examined as drug delivery platforms for MPTs; other delivery forms examined are noted in Tables 1, 2, 3.

**Table 3:** Summary of findings by delivery form, product attributes, and social factors.

Products and number of references	Vaginal administration 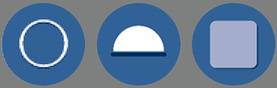	Oral administration 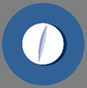	Injectable, implant, and microarray patch 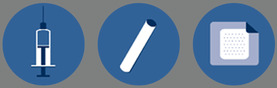
	Vaginal ring (*n* = 20), Gel (*n* = 7) with Diaphragm (*n* = 5), Film (*n* = 5), Other (*n* = 6)^a^	Oral tablet (*n* = 20)	Injectable (*n* = 15), implant (*n* = 5), patch (*n* = 3)
Product Attributes			
Type of Protection	Dual HIV and pregnancy prevention preferred for diaphragm with gel (23, 34).	When compared, greater importance was placed on HIV prevention efficacy vs. contraception efficacy (40, 46, 47)	Independently retrievable rods in an MPT implant was an appealing feature for end users, with some variation by site (45). End users preferred dual indication patches (27, 30, 28)
Familiarity	Unfamiliarity with vaginal dosing often led to initial hesitations (25, 31, 38, 40, 43, 47), which were overcome by counseling, information, and product use experience (18, 23, 31, 35).	Familiarity with tablets as a dosing form contributed to preference for MPT tablets (40, 43, 47, 51); among those with initial fears about tablets’ size or color, concerns decreased with product use experience (6, 38, 51).	Familiarity with injectables and implants as dosing forms contributed to preference (6, 18, 40, 43, 51) (45), although end users who had negative experiences with other injectables preferred non-injectable MPTs (40). Notably, the patch was an unfamiliar dosing form for all end users.
Discreetness	Hesitations about discreet use (38, 40) were overcome when end users found discreet use possible (35, 38) and made decisions around product use disclosure to partners (25, 38). Some viewed vaginal MPTs, particularly films and fabrics, as “woman initiated” and discreet (29, 31, 37, 39).	Although some felt tablets could be used without a partner’s knowledge (20, 21), discreet use was challenging due to a lack of privacy in the home to store and take pills and some expressed concerns that others would discover the pill bottle or raise concerns due to visual similarity between MPT tablet and ARVs (36, 38, 40, 50, 51).	Injectables were often preferred due to their heightened discreetness and ability to be used without partner detection (18, 38, 40, 51). The placement, flexibility/palpability, and biodegradability of an implant are important enablers of discreet use (45). High interest in the patch related to its potential for self-administration and discreet use (27, 28).
Frequency of Use	Opinions were varied on the acceptability of leaving a vaginal ring inserted for a month or longer (6, 35, 38, 40, 50, 51), as were preferences on dosing frequency and reasons for selecting other vaginally MPTs (29, 31, 39, 42).	Daily adherence was typically viewed as burdensome, particularly when taken at the same time each day, as were frequent clinic visits for tablet refills (6, 21, 40, 47, 50, 51). End users who valued lower frequency of use had lower preference for an MPT tablet (6, 39, 40, 50, 51).	Non-daily dosing was a positive attribute of injectable MPTs (18, 19, 38, 50, 51), implants (45) and patches (27, 28), although preferences for the ideal dosing interval varied widely (implants and injectables: 1 month–5 years; patch: 1–3 + months) (6, 18, 19, 27, 28, 30, 38, 40, 43, 45, 50, 51).
Side Effects	Side effects associated with an active MPT ring were assessed in one study (35) and with a placebo MPT ring in TRIO (6), as were concerns about side effects of a fabric MPT (31). Not discussed for diaphragms plus gel.	Despite some concerns about possible side effects (40, 47, 50, 51), tablets were typically perceived to have limited side effects and to be a safer delivery form because they could be stopped at any time (6, 18, 40, 50).	Although some end users expressed concerns about fear, pain, and side effects of injections (6, 38, 40, 50), these subsided after use experience (40). Similarly end users had concerns about pain with implant placement and removal (43, 45). End users wanted more information about patch side effects (30).
Fertility	Fertility was explored in one study of an active MPT ring, where end users expressed concerns about infertility caused by the ring that were related to rumors circulating in the community (35) Not discussed for other delivery forms.	Not discussed.	The potential for a separate, independently-removable contraceptive rod was highly salient for end users and return to fertility while maintaining HIV protection was of great interest (45).
Impact on Sex	Overwhelming preference for no change to the vaginal environment or interference with sex (31, 35, 37, 49, 51); specific preferences around changes to the vagina (e.g. wetness) were varied (26, 31, 39). End users that used rings and diaphragms during sex found them generally acceptable and rarely reported negative impacts (6, 23, 34, 35, 38, 51).	An MPT tablet's lack of interference with the sexual experience is an appealing feature to some end users (51).	The injectable MPT's lack of interference with sex was viewed as a positive feature for both women and their male partners (29, 51).
Delivery, Packaging, Messaging	End users desired marketing that emphasizes vaginal MPTs’ potential to empower women and enhance the sexual experience (6, 29, 34) but had mixed opinions on where diaphragm should be marketed (HIV vs. family planning) (31).	End users desired MPT tablets to be visually distinct from ARVs and had discreet, non-medical packaging (6, 20, 47). Potential channels of information for messaging included healthcare, traditional media, social media, and influencers (20).	End users and providers supported messaging that emphasizes both contraception and HIV indications, and that counseling at facilities should be augmented by community-level education and communication activities such as media and community-based awareness raising (45).
Social Factors			
Partners	Although partner-related social harms related to discovery of vaginal MPT use were minimal where reported (50), end users both anticipated and experienced resistance from male partners related to use of vaginal products (18, 31, 34, 35).	End users anticipated resistance and negative reactions from male partners if they discovered covert use of an MPT tablet, which could be mistaken for ARVs and/or indicate infidelity (20, 21, 36, 40, 51). Some felt that an MPT tablet could be an easier delivery form to “explain away” to a male partner compared to other forms (18, 40).	Male partners indicated that the increased discreetness of an MPT injectable and the dissimilarities with ARVs could be advantages for women with unsupportive or resistant partners (51). Similarly, implants placed in the same location as contraceptive implants could avoid partner detection as MPTs (45).
Healthcare Providers	End users expressed a strong desire for MPT ring and diaphragm counseling from providers and “testimonials” from other end users to support method uptake and use (23, 25, 47).	Health care providers saw both the potential benefits and implementation challenges of MPT tablets (21).	Health care providers may be critical and salient sources of information for education MPTs, although provider attitudes towards end users could influence uptake and use (29, 45). For implants, providers expressed preference for biodegrade, less flexible, palpable implants that were placed in the upper arm (45). Providers also supported the idea of independently retrievable rods for HIV protection during conception (45). Providers viewed patches as innovative with the potential to overcome issues related to daily adherence (27).
Community	Community members expressed and expected demand and support for MPT rings (22, 35), but end users anticipated community resistance related to norms around sex and contraception (31, 35).	End users anticipated stigma and judgment from the community due to presumptions that MPT tablet use indicated sexual promiscuity and mistaking MPT tablets for ARVs (6, 20, 21, 47, 51).	Policymakers felt that patches, like other MPTs, could address multiple sexual and reproductive health needs, and could alleviate workload in facilities with integrated service delivery (27, 28).

^a^ Other vaginally-administered products included insert (*n* = 3), hypothetical vaginal MPT (*n* = 2), and fabric (*n* = 1).

### 3.2. Product attribute factors

#### 3.2.1. Interest in MPTs for HIV and pregnancy prevention

Nearly every study assessed end users' preference for an MPT compared with single-indication products for HIV or pregnancy prevention. This yielded evidence of strong interest among reproductive-aged women for an MPT that simultaneously addresses HIV and pregnancy prevention (range across multi-country quantitative studies of 86%–93%). Participants also viewed MPTs as a product for improved sexual and reproductive health protection and reported that an MPT's overall purpose was more important than product-specific attributes (20, 23, 31, 34, 40, 46, 47, 52). Few studies reported reasons for not preferring MPTs; however, those that did noted the primary reason was a desire to conceive, retaining the option for flexibility, or concerns with drug toxicity (42, 45, 47, 52).

Preferences for the type of protection afforded by a dual-indication MPT product were mixed across studies. When TRIO participants were asked to select the one product attribute that most influenced their acceptability, almost half selected pregnancy prevention (44%) ahead of other factors. In other studies, participants placed more importance on HIV protection than pregnancy protection (20, 31, 39, 46, 47). Furthermore, across studies, participants noted the importance of having an HIV-only prevention option so that women would be able to continue protecting themselves against HIV when they want to have a child and would need to discontinue use of the MPT (44, 47, 52).

#### 3.2.2. Familiarity

Familiarity was an important acceptability factor across most studies that examined and compared specific delivery forms. Known and used delivery forms such as injectables and tablets were initially preferred and ranked higher than newer delivery forms such as the ring and implant (6, 18, 38, 40, 43, 44, 47, 50, 51). Reasons for preferring familiar products included decreased hesitation about side effects due to the ability to stop product use quickly, confidence in how to use the product discreetly, and ease of explanation to partners, family, peers, and community members (6, 18, 25, 38, 45, 50). However, initial concerns about unfamiliar products and unfamiliar product attributes could be overcome through learning about products and using products. For example, initial concerns over tablet color and size, and ring insertion and comfort, decreased after the opportunity to use placebo versions of these delivery forms. Similarly, concerns over vaginal insertion of a nanofiber fabric decreased after participants watched the product dissolve (6, 18, 31, 35, 38, 40, 51). Additionally, ratings and concerns for known and used products such as injectables and tablets changed minimally after demonstrations, educational videos, or actual use (6, 18, 38, 51), whereas increased exposure to and experience with novel delivery forms increased acceptability ratings and comfort (38, 39).

Participants' previous experience or lack of experience with family planning products also shaped preferences for MPT delivery forms (18, 29, 38–40, 43, 46, 50). For example, women who had previously used contraceptive implants or an IUD expressed a higher preference for the ring, women who had previously used birth control pills expressed a higher preference for the tablet, and women with only condom experience expressed a higher preference for films, inserts, and diaphragms (39, 50). Lastly, TRIO participants cited a lack of familiarity with new biomedical technologies as an important consideration with MPT introduction (42).

#### 3.2.3. Discreetness

Having the option to use a product discreetly was a key component of product acceptability among end users (6, 18, 27, 28, 30, 37, 38, 49, 52), some of whom described that their preferences for discreet products were driven primarily by concerns about a partner's inadvertent discovery of product use (40, 51) and potential disapproval (37). End users frequently noted that ideally they would like to talk to their partners about using an MPT (21) but that having the option of discreet use was essential because navigating discreet use or disclosing use to a partner was something unique to each individual and relationship (6, 25). Anticipated difficulties with discreet use were viewed as a substantial disadvantage (38, 40). Similarly, perceived ease of discreet use was a substantial driver of product preference (18, 29, 38, 40, 51). In one study, end users initially expressed concerns about partner detection of product use but later reported that this happened infrequently (38). Importantly, the physical delivery form of a product played a role in what discreet use could or might look like, with specific discretion-related considerations for each product; for example, physical location on the body and palpability of an implant (45). In a DCE with end users in South Africa, the importance of being able to use a product discreetly was rated with greater importance among end users who reported ever having difficulties negotiating condom use compared to those without condom negotiation difficulties (49).

#### 3.2.4. Frequency of administration and product duration

Frequency of administration or duration of use was a salient aspect of product acceptability for end users, and, when assessed, for their partners (26–28, 30, 40, 42, 51). Across studies and products, end users expressed a range of preferences for an ideal dosing frequency that most often ranged from 1 month to 1 year, with the ideal target duration varying by delivery form, study population, and location (6, 18, 19, 27, 28, 30, 32, 38, 40–43, 46, 50, 51). Preferences for ideal product duration were also often based on experience with HIV prevention or contraceptive products. For example, women who previously used long-acting contraceptives (i.e., implants and IUDs) often preferred long-acting MPTs, and women who previously used short-acting products (i.e., condoms) often preferred on-demand MPTs (39, 50).

Some end users described daily dosing regimens as burdensome or stressful and emphasized nondaily administration as a favorable attribute offering peace of mind and longer intervals of feeling “worry free” (18, 19, 38, 40, 50, 51). They also noted potential adherence challenges with daily dosing regimens, describing that daily stressors or unexpected events could interfere with routines (40, 51). Other end users raised concerns about long-acting products with infrequent dosing such as forgetting to re-administer products at the appropriate time, particularly user-controlled methods that required vaginal insertion monthly (6), and unknown health impact of long-acting product use (6, 51). A smaller proportion of end users noted that event-driven dosing was an appealing option for people who engaged in infrequent sexual activity (29, 42). End users who engaged in vaginal sex more frequently had lower preference for a product administered before sex, whereas end users who engaged in less frequent sex had lower preference for a product administered daily (26).

#### 3.2.5. Side effects

End user perceptions of, and experiences with, side effects such as pain and menstruation were varied. The available data indicated that although some end users had concerns about side effects of potential active MPTs, most end users discussed pain and discomfort with product administration more frequently and saliently than drug-related side effects. For example, end users discussed fear of painful MPT placement or administration within research about injectables, implants, and rings (6, 30, 43, 45).

Overwhelmingly, end users preferred products that did not alter their menstrual cycles (6, 29, 35, 40, 42), although some preferred lighter menses (39). Additionally, end users had mixed opinions about using a vaginally-administered product during menstruation, with some noting a dislike of the idea of inserting a product while menstruating; others had concerns about product displacement or reduced efficacy during menstruation (25, 31). Additionally, end user concerns about drug-related side effects were minimal but were mentioned by end users in research related to tablets and the microarray patch (30, 51). In a market research study with women in Uganda, Nigeria, and South Africa, country-level differences were found in tolerance of side effects, with more participants in Uganda finding a wide range of side effects (e.g., migraines, menstrual irregularities, nausea) to be unacceptable compared with participants in South Africa and Nigeria (29).

#### 3.2.6. Fertility

Effects of MPT use on fertility and product-related preferences to facilitate return to fertility were explored infrequently in the reviewed articles. This topic was largely examined within studies on nanofiber fabric and implants and constituted one of the attributes included in MTN 045/CUPID, which included vaginal film/inserts, vaginal ring, and oral tablets (24, 29, 31, 42, 45). Some end users expressed preferences for MPT products that allowed for flexibility in contraception administration or similarly noted that lack of flexibility in contraception coverage was a limitation of specific methods (31, 45). For example, end users were highly interested in an MPT implant with a distinct contraceptive implant component that could be removed in the event of a desire to return to fertility. Some end users expressed concerns about long-term MPT use affecting fertility and fetal development (52). Overall, a range of preferences (immediate, 3 months, 6 months) regarding return to fertility following product discontinuation were found in MTN 045/CUPID, with this attribute not significantly influencing product choices. Zimbabwean women preferred a more immediate return to fertility as compared with Ugandan women who regarded a longer return to fertility as an extended benefit of the product following discontinuation (42).

#### 3.2.7. Impact on sex

Across most studies, female participants revealed a preference for products that did not interfere with sex or sexual pleasure for their male partners (6, 23, 24, 31, 34, 37, 38, 40, 51). Consequently, participants were initially disinterested in products (ring, diaphragm, fabric) that would be inserted into the vagina, could potentially change vaginal dryness or wetness, or become dislodged during sex. However, acceptability and ratings for vaginally inserted products increased after participants had the opportunity to learn more about the product or try the product (6, 23, 24, 31, 42, 51). Lack of interference with sex was described as a positive attribute for products (injectable, tablet) that could be taken before an encounter as they would make participants feel prepared and limit the opportunity for partners to notice or stop product use (29, 51). The effects of an MPT product on the sexual experience was explored extensively in studies where women used study products serving as MPT proxies, such as placebo versions in TRIO, and in research on the diaphragm and gel (23, 34). The impact on sex was explored minimally in relation to the nanofiber fabric and in non-TRIO general MPT research.

Overwhelmingly, end users preferred products that improved the sexual experience, did not alter the vaginal environment, or did not interfere with sex (6, 31), a sentiment echoed among end users' male partners (24, 51). Similarly, the expected or actual interference with sex was described as a barrier to product acceptability and use (37), whereas perceiving a product to have a limited influence on sex was associated with more favorable overall acceptability ratings (34, 38). However, some variations in preferences were found by country setting. For example, MTN-045/CUPID found that while participants in Zimbabwe preferred products that did not influence the vaginal environment, participants in Uganda preferred a product that increased vaginal wetness during sex (42).

#### 3.2.8. Delivery, packaging, messaging

Few studies examined end user's preferences for MPT distribution and delivery. In studies that did examine this question, women generally indicated a preference for receiving MPT products through a government health facility or with an official prescription (25, 32). Additionally, when asked to select the one attribute that most influenced acceptability in research with former TRIO participants and product-naïve end users, almost one-quarter of participants selected distribution location (40). Participants reported that over-the-counter availability would increase MPT acceptability and uptake and that education and information on MPT product options should be readily available at health clinics to be integrated into contraceptive and HIV prevention decisions (29, 45). In qualitative research with TRIO participants, end users emphasized the importance of community sensitization and dispelling misperceptions about MPTs as essential components of MPT introduction (6). End users also called for opportunities to try MPT delivery forms, particularly those that may be novel, before deciding to use a particular product (6).

Among studies reporting on design and packing preferences for MPTs, participants suggested “feminine” or “sexy” packaging to make MPTs look appealing, similar to existing branding approaches for menstrual products (6). A few studies stressed the importance of packaging being discreet, small, and nonmedical, such as face powder, chocolate box, lip gloss tube, or snuff boxes (6, 20, 47). The nonmedical preference was particularly important for tablets because participants wanted to avoid the stigma of MPT tablets being confused with ARV tablets (6, 20, 47). Opinions were mixed on whether MPTs should equally emphasize pregnancy and HIV prevention in their packaging, rather than only one indication. Some participants believed that emphasizing only pregnancy prevention might be more discreet, amenable for wary male partners, and a way to avoid HIV-related stigma or assumptions of infidelity (20, 24, 45, 47).

Participants suggested several MPT benefits to emphasize in future MPT messaging, including dual protection, women's empowerment, enhanced sexual pleasure, and increased safety and control over sexual and reproductive health for women (6, 20, 29, 47). Community sensitization was reported as essential for the rollout of any future MPT product to dispel misperceptions about MPTs and for individuals to ask questions (6, 18, 20, 45). One study specifically noted that for an MPT to be acceptable in the community and within relationships, it must be available for everyone, and it must be extremely public, which is similar to the rollout of voluntary male medical circumcision (20).

### 3.3. Social factors findings

#### 3.3.1. Partners

Women's views of male partner MPT acceptability varied across studies. In some studies, participants were hesitant to use MPTs because of potential negative reactions from male partners and the potential impact on men's sexual pleasure. Expectations of negative reactions were based on previous negative experiences in disclosure of HIV prevention or contraceptive use and a preference to avoid conversations about HIV prevention. In some instances, male partners were distrustful of their partners for concealing or delaying disclosure of study participation or they assumed that using HIV prevention methods meant the female partner was promiscuous and engaging in other sexual relationships (6, 20, 21, 24, 36, 40, 50, 51). Participants also were wary that male partners would not approve of vaginally inserted products or products that interrupted the sexual encounter because they might change the vaginal environment and decrease sexual pleasure for men (6, 24, 31, 39–42). Some participants indicated that negotiating MPT use with male partners may be easier than negotiating use of separate HIV and pregnancy prevention methods, particularly if they could omit the HIV prevention benefits component with MPTs (24, 52). Additionally, participants noted that it would be easier to explain away MPTs with known delivery forms such as a tablet or injectable, as compared with novel MPT delivery forms, such as the ring, implant, fabric, or insert (18, 40). Despite these concerns regarding disclosure, and a preference for a product that a partner would not notice during sex, women commonly indicated that they would tell a primary partner they were using a product even if it could be used without partner detection; as found, for example, among two-thirds of women in Zimbabwe and South Africa participating in the Quatro study (26).

Male partner's views on MPT acceptability also varied across studies. Some male partners could acknowledge the benefits of MPTs for HIV and pregnancy protection but were concerned with limiting potential MPT side effects that impacted sexual pleasure (such as vaginally inserted products and changes in menstruation and wetness) and female partners using products discreetly (24, 42, 51). Other male partners were supportive of women using MPTs and acknowledged the personal benefits of MPTs to them, expressed concern about product adherence, and had more positive views of products that women could more easily use with consistency (24, 51). Participants enrolled in a couples MPT study described that the process of discussing and selecting a hypothetical ideal product together as a couple resulted in greater satisfaction with their chosen product because it built trust and communication and allowed individuals to focus on the interests of the couple over that of the individual (24, 42).

#### 3.3.2. Healthcare providers

End user perspectives on healthcare provider impact on MPT acceptability was infrequently assessed. Health care providers were generally seen as an important and trusted source of information, although there were some region-level differences in these perspectives (29). For novel or unfamiliar products, end users expressed a strong desire for counseling from health care providers to ensure they received adequate support on product administration and use (25, 47). For products designed to be user-controlled or that could be self-administered, such as the microarray patch, women considered self-administration acceptable and expressed a desire to first receive instruction from a health care provider (30). Some end users expressed concerns about health care providers' stigmatizing attitudes toward those who used MPTs, particularly young women and married people (21, 25, 45).

Health care provider perspectives on MPT products were frequently product specific. However, providers generally expressed positive attitudes toward MPTs and perceived them as innovative approaches that could empower women, reduce unplanned pregnancies, and reduce new HIV infections in their communities (21, 27, 33). In considering health systems factors, health care providers noted that MPTs could provide efficiencies in reducing frequency of clinic visits and improving accessibility (21). Some providers noted advantages of reduced burden in frequency of women's interactions with the healthcare system tied to use of self-administered delivery forms like the microarray patch and long-acting delivery forms such as implants (30, 45). However, other providers noted that regulatory requirements could mean that products may only be available in regulated dispensaries, which could reduce accessibility (25).

#### 3.3.3. Community stakeholders

Few studies examined how community stakeholders impacted MPT acceptability and uptake potential. Stakeholders and policymakers acknowledged the benefits of overall MPTs and reported that their development (such as the ring or patch) could be particularly useful for AGYW (22, 28). Some participants were wary of the potential HIV-related and sexual activity related stigma that would coincide with using an MPT product (such as a tablet or diaphragm), particularly if it looked like ARV medication or was advertised as an HIV prevention product rather than a dual-indication product or pregnancy prevention product (6, 20, 21, 34, 47, 51). Participants and providers both suggested community sensitization and provider forum sessions to decrease MPT-related fears and stigma, particularly among men (6, 18, 20, 45). In one study, some participants noted that religious prohibition of the use of contraception could be a potential barrier in their communities to fabric acceptance and uptake (31).

## 4. Discussion

The present scoping review synthesizes existing research on MPTs that was conducted amongst women of reproductive age in SSA and their male partners, healthcare providers, and community stakeholders. The aim of the review was to identify factors that are important for optimizing the likelihood of MPT acceptability and future adoption by end users in the region. Overall, there was a strong interest amongst women and healthcare providers for an MPT that simultaneously addresses HIV and pregnancy prevention. However, due to changing reproductive needs throughout the life course, women valued MPTs as an additional option to add to the existing (and growing) range of HIV and pregnancy prevention options. Though women and health care providers often preferred long-acting MPTs, there was considerable variation by product familiarity and form, as well as study population. Unfamiliarity with novel delivery forms, particularly with forms that were vaginally administered, was an initial barrier across most studies but was often addressable through counseling and experience trying a product. The ability to use an MPT discreetly – through its physical design, attributes, and administration—was one of the most salient topics for end users and was more frequently examined in the existing literature compared to other MPT factors such as side effects, fertility, and impact on sex. Importantly, current knowledge about end user preferences for MPTs is largely based on end user experience with placebo or hypothetical MPT products and there is potential for MPT acceptability, attitudes, and adoption experiences to considerably vary after end users have access to active MPT products and experience side effects tied to each indication.

The integration of HIV prevention and contraceptive services that an MPT could afford was cited by women and health care providers as a critical advantage. Healthcare providers reported that MPTs could potentially provide efficiencies in reducing clinic burden, frequency of clinic visits, and adherence challenges among women. End-users indicated a strong preference for MPTs to be available through family planning service settings to de-medicalize HIV prevention. Several studies have highlighted the importance of examining models to achieve this through dual provision of existing HIV and pregnancy prevention services such as HIV testing, PrEP, and contraception (55). However, implementation science-oriented evidence relevant to integration of MPTs into health delivery systems is sparse (e.g., training needs, cost, and effective counseling and decision-making models for end-users, the male partners, and their community members) (56). Future research to explore these domains is necessary not only for eventual MPT delivery but also for dual delivery of existing single indication prevention options.

In general, most women preferred longer-acting MPTs (one month or more, depending on delivery form), because they were perceived to reduce user dosing burden and allow for more discreet use. This finding aligns with SSA-based studies that have reported adherence challenges with daily use of oral PrEP (57, 58) and was echoed in the Share.Learn.Shape study that indicated increased interest in long-acting methods (specifically implant, ring and injection) among women in low- and middle-income countries compared with those from high-income countries (59). Providers likewise recognized advantages of longer-acting MPT options in reducing demands on the health care system; however, research with providers is limited and largely drawn from small qualitative studies. The classification of “longer-acting” was conceptualized differently depending on whether products were delivered vaginally or via implant. Yet, the longest duration examined, was often, but not always, the most preferred. In many studies there consistently remained a subset of women with an interest in on-demand MPT options that afforded user control and flexibility. The contraceptive model of providing a method mix with provider-administered longer-acting reversible contraceptives alongside user-delivered, shorter-acting methods has been important in increasing family planning product adoption and use (60). The model also offers a uniquely relevant and compelling strategy for conceptualizing development of multiple MPT options.

Familiarity with the MPT delivery form prominently influenced initial acceptability with the strongest evidence derived from DCE and placebo clinical studies. This was particularly evident in the preference for injectables among those with injectable contraceptive experience. A review of values and preferences informing contraceptive use highlighted a similar finding that familiarity was a primary factor in decision-making among contraceptive options (61). However, multiple clinical studies signaled that lack of familiarity can be addressed and, importantly remained an interest in new delivery forms across studies (29, 40, 42). Both the TRIO and Quatro MPT and HIV placebo clinical studies underscored that with increased opportunity to use and gain experience with novel vaginally-administered products, acceptability ratings for products increased over time (38, 54). User experience with placebo microneedle patch likewise increased acceptability of an otherwise unfamiliar MPT delivery form (30). Research focused exclusively on HIV prevention also reflects the influence of use experience on increasing acceptability; in the REACH Study, two-thirds of adolescent girls and young women chose to use the dapivirine vaginal ring (an initially unfamiliar product) for HIV prevention after using the ring and oral PrEP for six months each (62). Taken collectively, familiarity with delivery form may facilitate earlier adoption for many women but education and use experience can increase acceptability for novel delivery forms.

An important partner-related consideration is how an MPT may help women overcome male partners' resistance to their use of an HIV prevention product by positioning the method as a contraceptive, first and foremost, and de-emphasizing implications of sexual fidelity and risk behavior. This consideration was infrequently examined as was the degree to which the availability of a range of MPTs will increase adoption or influence use of contraceptive methods. However, women in MTN 045/CUPID noted these advantages as did health care providers in TRIO, pointing towards the importance of marketing and communications materials related to MPTs. In several other studies, women reported that MPT packaging should emphasize pregnancy prevention instead of HIV, for acceptability reasons associated with privacy and discretion to partners and other individuals in their social network (20, 45, 47). Across the MPT research, whether conducted with women alone, or those that included men and male partners, there is strong evidence of the important role that partners assume in shaping women's MPT preferences and acceptability by indirectly influencing women's perceptions of product attributes and directly influencing women's decision-making. For women coupled with casual or unsupportive partners, potential use of MPTs without a partner's detection was regarded as valuable, and products with non-daily dosing, clinic-based administration, and undetectability during sex were important as their characteristics might contribute to this goal. Including opportunities for male partner involvement in MPT development and delivery, while preserving women's agency to use products independently, may ultimately address many of the discreetness considerations and increase MPT adoption.

Given that most MPTs in the pipeline are in pre-clinical development, most studies assessed preferences through presentation of hypothetical product descriptions, images, or product models. While the existing body of research offers important findings to inform early product development and to iterate designs, very few studies report on research in which women used placebo or active MPT products. This evidence base reflects the state of the field where few MPT products have yet been evaluated in clinical studies. Although preferences derived through DCEs have been shown in other areas of health research to correlate with choices among actual prevention options (63), the extent to which the findings synthesized in this review will ultimately reflect end users' actual use experiences and the trade-offs they may be willing to make to achieve dual protection with an active MPT product is unknown. Thus, it remains important to include robust social behavioral and end-user research as part of the MPT research agenda, particularly to conduct studies with novel placebo delivery forms to refine their design and understand user experiences and factors influential to acceptability of new MPT products, particularly related to side effects. Research with active pharmaceutical ingredients (API), be they with contraceptive or HIV prevention indications, provide strong evidence for the importance of the impact of side effects on user experience and acceptability. Side effects, whether actual or perceived, are often a primary reason for contraceptive method switching (64). For example, in a cohort study examining contraceptive discontinuation and switching among Kenyan women, lack of expected menstrual bleeding was associated with method switching and multiple side effects, including sexual side effects, irregular bleeding, weight changes, and increased rates of method discontinuation (65). Thus, although several studies included in this review provided evidence that side effects were important to women's preferences, we anticipate that side effects and implications on timing of return to fertility could emerge as more important factors when MPT products are examined in clinical trials. Likewise, given the importance of discretion, examining whether and how women are able to use products discreetly, will be critical as we move from hypothetical studies to clinical trials of MPT products and ultimately MPT introduction.

The literature synthesized for this review has several important limitations and gaps. First and foremost, the breadth and rigor of the available research on end-user preferences for single indication HIV and pregnancy prevention options are abundant, but sparse when specifically about dual indication MPTs. Despite extending our search to include conference abstracts, grey literature reports, unpublished research obtained through personal communication with subject matter experts, and research databases—our review yielded only 37 references. Furthermore, many of our references (59%) reported results of qualitative research where hypothetical or placebo MPT options were considered, and a substantial proportion of the of the articles (30%) reported data from the TRIO study. Second, the generalizability of findings must consider the heterogeneity of women in the SSA region. Most of the evidence in this review comes from end users in South Africa, Kenya, and, to a lesser extent, Zimbabwe. In addition, the majority of studies were conducted in urban or peri-urban areas and included women who would be most likely to access care in public health and research clinic settings, resulting in very limited perspectives from end users living in peri-urban and rural areas and other countries in SSA. Further, women who join research studies, and studies that cover novel biomedical methods may have different individual- and relationship-level characteristics than those who do not enroll. In addition, few studies included cross-country comparisons. The lack of diversity in research populations and settings, and limited cross-country comparisons, warrants careful consideration of the end users that have contributed to this evidence, and the broader potential populations of MPT users across sub-Saharan Africa. It also highlights the importance of conducting multisite and multi-country clinical trials and research studies for future active MPT products. Third, most of the peer-reviewed and grey literature is focused on overall acceptability of MPTs. Based on frequency of mentions in this literature, discretion and partner engagement are salient considerations to MPT acceptability, and findings echo those from HIV prevention and contraceptive choice research. Additionally, acceptability is a nuanced construct to assess in end-user research with MPTs. This is due to an array of factors including the diversity of end user experiences, lack of consensus on how to best assess acceptability, and nuanced relationships between acceptability and compliance and adherence. In a clinical trial setting, acceptability data are also subject to social desirability bias, and to complexities whereby an “acceptable” product in a trial setting may not translate to a product that end-users will prefer and use consistently in a real-world circumstance. However, there remains opportunity to further consider how to effectively engage men and couples throughout the MPT product development pipeline. MPTs' impacts on sex, including on sexual pleasure, are explored to some extent, although more research, with actual and placebo delivery forms, may be needed to understand the diversity of end user preferences. Very little research has been conducted with providers and other community stakeholders, limiting our ability to characterize their views in a rigorous and substantive manner.

## 5. Conclusion

The present scoping review of end-user preferences and acceptability for MPTs underscores women's strong interest in MPTs and the importance of multiple MPT options. Recognizing the heterogeneity of women's preferences, and within women, changing needs for HIV and pregnancy prevention over their reproductive life course and relationships, the central concept of “choice” should be understood and integrated in multiple ways. For example, choice includes offering MPTs within delivery of family planning and HIV prevention services, as well as choice among MPTs with distinct product profiles. However, current knowledge about end user preferences for MPTs is largely based on end user experience with existing single indication HIV prevention and contraceptives or studies that used placebo or hypothetical MPT products. Conducting research where end user experience with active products can be evaluated stands to advance understanding of end-user preferences and acceptability for MPTs.

## References

[1] Bearak JM Popinchalk A Beavin C Ganatra B Moller AB Tuncalp O Country-specific estimates of unintended pregnancy and abortion incidence: a global comparative analysis of levels in 2015–2019. BMJ Glob Health. (2022) 7(3):e007151. 10.1136/bmjgh-2021-007151 35332057 PMC8943721

[2] Zhang J Ma B Han X Ding S Li Y. Global, regional, and national burdens of HIV and other sexually transmitted infections in adolescents and young adults aged 10-24 years from 1990 to 2019: a trend analysis based on the global burden of disease study 2019. Lancet Child Adolesc Health. (2022) 6(11):763–76. 10.1016/S2352-4642(22)00219-X 36108664

[3] Consolidated guidelines on HIV prevention, testing, treatment, service delivery and monitoring: Recommendations for a public health approach. Geneva: World Health Organization (2021). 34370423

[4] Tolley EE Morrow KM Owen DH. Designing a multipurpose technology for acceptability and adherence. Antiviral Res. (2013) 100(Suppl(0)):S54–9. 10.1016/j.antiviral.2013.09.029 24188706 PMC4643455

[5] Young IC Benhabbour SR. Multipurpose prevention technologies: oral, parenteral, and vaginal dosage forms for prevention of HIV/STIs and unplanned pregnancy. Polymers (Basel). (2021) 13(15):2450. 10.3390/polym13152450 34372059 PMC8347890

[6] Minnis AM Krogstad E Shapley-Quinn MK Agot K Ahmed K Danielle Wagner L Giving voice to the end-user: input on multipurpose prevention technologies from the perspectives of young women in Kenya and South Africa. Sex Reprod Health Matters. (2021) 29(1):1927477. 10.1080/26410397.2021.1927477 34224341 PMC8259853

[7] Boonstra H Barot S Lusti-Narasimhan M. Making the case for multipurpose prevention technologies: the socio-epidemiological rationale. BJOG. (2014) 121(Suppl 5):23–6. 10.1111/1471-0528.12851 25335837

[8] Sully EA Biddlecom A Darroch JE Riley T Ashford LS Lice-Deroche N Adding it up: Investing in sexual and reproductive health 2019. New York: Guttmacher Institute (2020).

[9] Crankshaw TL Smit JA Beksinska ME. Placing contraception at the centre of the HIV prevention agenda. Afr J AIDS Res. (2016) 15(2):157–62. 10.2989/16085906.2016.1204330 27399045

[10] MPT Product Development Database. The Initiative for Multipurpose Prevention Technologies. Available from: http://mpts101.org/ (cited April 26, 2023).

[11] Young Holt B Moore S Hemmerling A Nanda K Kopf G Palanee T Multipurpose prevention technologies: strategy recommendations to guide the most promising products from the lab to hands of women. J Int AIDS Soc. [Conference Abstract]. (2021) 24(Suppl 1):144. 10.1002/jia2.25659

[12] Krovi SA Johnson LM Luecke E Achilles SL van der Straten A. Advances in long-acting injectables, implants, and vaginal rings for contraception and HIV prevention. Adv Drug Deliv Rev. (2021) 176:113849. 10.1016/j.addr.2021.113849 34186143

[13] Chakhtoura NC. Multipurpose prevention technologies (MPTs) for prevention of HIV and pregnancy. HIV Research for Prevention (R4P); Virtual (2021).

[14] Friedland BA Mathur S Haddad LB. The Promise of the Dual Prevention Pill: A Framework for Development and Introduction. Front Reprod Health. (2021). 10.3389/frph.2021.682689 PMC8312733 34318291

[15] Witte SS Filippone P Ssewamala FM Nabunya P Bahar OS Mayo-Wilson LJ PrEP acceptability and initiation among women engaged in sex work in Uganda: implications for HIV prevention. EClinicalMedicine. (2022) 44:101278. 10.1016/j.eclinm.2022.101278 35128367 PMC8808048

[16] Munn Z Peters MDJ Stern C Tufanaru C McArthur A Aromataris E. Systematic review or scoping review? Guidance for authors when choosing between a systematic or scoping review approach. BMC Med Res Methodol. (2018) 18(1):143. 10.1186/s12874-018-0611-x 30453902 PMC6245623

[17] Mensch BS van der Straten A Katzen LL. Acceptability in microbicide and PrEP trials: current status and a reconceptualization. Curr Opin HIV AIDS. (2012) 7(6):534–41. 10.1097/COH.0b013e3283590632 23032737 PMC4026162

[18] Agot K Minnis AM Manenzhe K Browne EN Ahmed K Okello T Engaging study participants in interpreting results: lessons from the TRIO study in Kenya and South Africa. Int J Womens Health. (2019) 11:395–403. 10.2147/IJWH.S193905 31372060 PMC6636186

[19] Agot K Lutnick A Shapley-Quinn MK Ahmed K Okello T van der Straten A. “I felt like a TRIO champion”: end-user perspectives on their role as co-designers of multi-purpose technologies. Gates Open Res. (2020) 4:163. 10.12688/gatesopenres.13182.1 33870103 PMC8028846

[20] AVAC. Dual prevention pill: The Eureka Workshops recommendations and solutions (2021).

[21] Barker T Rodrigues J, editors. Bringing the dual prevention pill to market: opportunities for HIV and pregnancy prevention and implications for future multipurpose prevention technologies (MPTs). Berlin: IAS (2021).

[22] Bayigga J Onyango M Nassuuna I Kusemererwa S, editors. Need for a multi-purpose dapivirine vaginal ring to address sexual and reproductive health challenges: lessons learnt from South Western Uganda. HIV Research for prevention (HIVR4P) conference; 2018; Madrid, Spain: international AIDS society.

[23] Beksinska M Greener R Smit J Maphumulo B Mphili N Kilbourne-Brook M A randomized crossover study evaluating the use and acceptability of the SILCS diaphragm compared to vaginal applicators for vaginal gel delivery. AIDS Behav. (2018) 22(1):127–32. 10.1007/s10461-017-1913-4 28993940

[24] Bhushan NL Musara P Hartmann M Stoner MCD Shah SR Nabukeera J Making the case for joint decision-making in future multipurpose prevention technology (MPT) choice: qualitative findings on MPT attribute preferences from the CUPID study (MTN-045). J Int AIDS Soc. (2022) 25(10):e26024. 10.1002/jia2.26024 36254362 PMC9577116

[25] Bowen J Lech D Matsumoto S Roane A. Exploring Intravaginal Ring Acceptability for Disease Prevention Among At-Risk Community Members in Cape Town. (2017).

[26] Browne EN Montgomery ET Mansfield C Boeri M Mange B Beksinska M Efficacy is not everything: eliciting Women’s Preferences for a vaginal HIV prevention product using a discrete-choice experiment. AIDS Behav. (2020) 24(5):1443–51. 10.1007/s10461-019-02715-1 31696371 PMC6990865

[27] Gachigua GS Karuga R Ngunjiri A Jarrahian C Kilbourne-Brook M Otiso L, editors. “It is cool; once you place it, that is it": exploring the acceptability, usability, and programmatic fit of microarray patches (MAP) as multipurpose technology (MPT) for prevention of both HIV and unintended pregnancy among adolescent girls in Kenya. International workshop on HIV & adolescence; 2022; Cape Town, South Africa.

[28] Gachigua GS Karuga R Ngunjiri A Jarrahian C Kilbourne-Brook M Otiso L. Acceptability and usability of microarray patch for HIV prevention and as a multipurpose prevention technology to protect from HIV and unintended pregnancy in Kenya: “killing two birds with one stone”. [Abstract for submission to frontiers in reproductive health] (In press). 10.3389/frph.2023.1125159 PMC10164997 37168102

[29] Ipsos. MPT Acceptability in Uganda, Nigeria and South Africa understanding the women, the End user. Ipsos Healthcare (2014). https://theimpt.org/mpt-product-development-regulatory-issues-102

[30] Kilbourne-Brook M Ismail A Magni S Fellows T Ruhweza Katahoire A Ayebare F User assessment of a microarray patch for HIV PrEP and as a multipurpose prevention technology for HIV and pregnancy prevention: perspectives from Uganda and South Africa. J Int AIDS Soc. [Conference Abtract]. (2021) 24(Suppl 1):10–1. 10.1002/jia2.25659

[31] Laborde ND Leslie J Krogstad E Morar N Mutero P Etima J Perceptions of the “fabric”—an exploratory study of a novel multi-purpose technology among women in sub Saharan Africa. PLoS One. (2018) 13(10):e0204821. 10.1371/journal.pone.0204821 30379839 PMC6209182

[32] Lunani LL Namukwaya S Lipesa S Kombo B Omondi D Shikuku J, editors. Perspectives and preferences for multi-purpose prevention technologies (MPTs) to address sexual and reproductive health (SRH) needs among adolescent girls and young women (AGYW) in Kenya and Uganda. International AIDS society (IAS) conference; 2022; Montreal, Canada.

[33] Lutnick A Shapley-Quinn MK Manenzhe KN Onyango J Agot K Ahmed K Two birds with one stone: health care Providers’ perspectives about prevention technologies in Kenya and South Africa. J Int Assoc Provid AIDS Care. (2019) 18:2325958219841366. 10.1177/2325958219841366 31018754 PMC6748465

[34] MatCH Research Unit. Assessment of opportunities and challenges for potential introduction of the SILCS diaphragm in South Africa (2016).

[35] McLellan-Lemal E Deaton SR Betts JE Ondenge K Mudhune V O’Connor SM Acceptability of an intravaginal ring for simultaneously preventing HIV infection and pregnancy: qualitative findings of the kisumu combined ring study, 2019. Contemp Clin Trials. (2022) 122:106935. 10.1016/j.cct.2022.106935 36162740 PMC11265295

[36] Mgodi N Rodrigues J, editors. Demand, delivery, and data for decision-making: How market preparation for the Dual Prevention Pill is reimagining prevention programs for a future with MPTs. AIDS; 2022 July 31, 2022; Montreal, Canada and virtually.

[37] Milford C Rambally L Kubeka M Moore L Beksinska M Kilbourne-Brook M Introduction of the SILCS diaphragm as a multipurpose technology in South Africa: potential users, perceived benefits, and barriers to use. Aids Res Hum Retrovir. [Meeting Abstract]. (2014) 30:A67-A. 10.1089/aid.2014.5122.abstract

[38] Minnis AM Roberts ST Agot K Weinrib R Ahmed K Manenzhe K Young women’s ratings of three placebo multipurpose prevention technologies for HIV and pregnancy prevention in a randomized, cross-over study in Kenya and South Africa. AIDS Behav. (2018) 22(8):2662–73. 10.1007/s10461-018-2078-5 29560570 PMC6097726

[39] Minnis AM Montgomery ET Napierala S Browne EN van der Straten A. Insights for implementation science from 2 multiphased studies with end-users of potential multipurpose prevention technology and HIV prevention products. J Acquir Immune Defic Syndr. (2019) 82(Suppl 3):S222–9. 10.1097/QAI.0000000000002215 31764258

[40] Shapley-Quinn MK Manenzhe KN Agot K Minnis AM van der Straten A. “We are not the same”: African women’s view of multipurpose prevention products in the TRIO clinical study. Int J Womens Health. (2019) 11:97–107. 10.2147/IJWH.S185712 30799959 PMC6369839

[41] Minnis AM Browne EN Boeri M Agot K van der Straten A Ahmed K Young women's stated preferences for biomedical HIV prevention: results of a discrete choice experiment in Kenya and South Africa. J Acquir Immune Defic Syndr. (2019) 80(4):394–403. 10.1097/QAI.0000000000001945 30633040 PMC6410963

[42] Minnis AM Etima J Musara P Browne EN Mutero P Kemigisha D Couples’ preferences for “2 in 1” multipurpose prevention technologies to prevent both HIV and pregnancy: results of a discrete choice experiment in Uganda and Zimbabwe. AIDS Behav. (2022) 26(12):3848–61. 10.1007/s10461-022-03713-6 35674885 PMC9175528

[43] Namukwaya S Katumba K Kayesu I Nabalwanyi Z Naluwooza R Mayanja Y, editors. Female sex workers’ preferences for multi-purpose technologies to prevent HIV, other sexually transmitted infections and unintended pregnancies in Kampala, Uganda. International AIDS society (IAS conference; 2022; Montreal, Canada).

[44] Nkomo S Mahaka I Luecke E Van Der Straten A Shapley-Quinn MK Makoni W, editors. End-users’ hypothetical acceptability of a biodegradable implant to prevent HIV and unplanned pregnancy: qualitative insights from South Africa and Zimbabwe. International AIDS society (IAS), HIV research for prevention (HIV4RP) virtual conference (2021).

[45] Nkomo S Makoni W Shapley-Quinn MK Luecke E Mbatsane E Manenzhe K Prospective acceptability of a multipurpose technology (MPT) implant in preclinical development to prevent HIV and unplanned pregnancy: qualitative insights from women End users and health care providers in South Africa and Zimbabwe. PLoS ONE. Under review. 10.1371/journal.pone.0285711 PMC10191314 37195918

[46] Quaife M Eakle R Cabrera Escobar MA Vickerman P Kilbourne-Brook M Mvundura M Divergent preferences for HIV prevention: a discrete choice experiment for multipurpose HIV prevention products in South Africa. Med Decis Making. (2018) 38(1):120–33. 10.1177/0272989X17729376 28863752

[47] Routes2Results. Understanding consumer preference for HIV prevention products: Quantitative findings from surveys with 18–21 year old young women in South Africa. Routes2Results (2017).

[48] Stoner MCD Browne EN Etima J Musara P Hartmann M Shapley-Quinn MK Couples’ decision making regarding the use of multipurpose prevention technology (MPT) for pregnancy and HIV prevention. AIDS Behav. (2023) 27(1):198–207. 10.1007/s10461-022-03756-9 35776249 PMC9805468

[49] Terris-Prestholt F Hanson K MacPhail C Vickerman P Rees H Watts C. How much demand for new HIV prevention technologies can we really expect? Results from a discrete choice experiment in South Africa. PLoS One. (2013) 8(12):e83193. 10.1371/journal.pone.0083193 24386160 PMC3875434

[50] Weinrib R Minnis A Agot K Ahmed K Owino F Manenzhe K End-users’ product preference across three multipurpose prevention technology delivery forms: baseline results from young women in Kenya and South Africa. AIDS Behav. (2018) 22(1):133–45. 10.1007/s10461-017-1911-6 29052018 PMC5758675

[51] Wagner LD Minnis AM Shea J Agot K Ahmed K van der Straten A. Female and male partner perspectives on placebo multipurpose prevention technologies (MPTs) used by women in the TRIO study in South Africa and Kenya. PLoS One. (2022) 17(5):e0265303. 10.1371/journal.pone.0265303 35551318 PMC9097999

[52] Woodsong C Musara P Chandipwisa A Montgomery E Alleman P Chirenje M Interest in multipurpose prevention of HIV and pregnancy: perspectives of women, men, health professionals and community stakeholders in two vaginal gel studies in Southern Africa. BJOG. (2014) 121(Suppl 5):45–52. 10.1111/1471-0528.12875 25335840

[53] van der Straten A Agot K Ahmed K Weinrib R Browne EN Manenzhe K The tablets, ring, injections as options (TRIO) study: what young African women chose and used for future HIV and pregnancy prevention. J Int AIDS Soc. (2018) 21(3):e25094. 10.1002/jia2.25094 29600595 PMC5876496

[54] Montgomery ET Beksinska M Mgodi N Schwartz J Weinrib R Browne EN End-user preference for and choice of four vaginally delivered HIV prevention methods among young women in South Africa and Zimbabwe: the quatro clinical crossover study. J Int AIDS Soc. (2019) 22(5):e25283. 10.1002/jia2.25283 31069957 PMC6506690

[55] Nyaboe E Larsen A Sila J Kinuthia J Owiti G Abuna F Contraceptive method mix and HIV risk behaviors among Kenyan adolescent girls and young women seeking family planning services: implications for integrating HIV prevention. Front Reprod Health. (2021) 3:667413. 10.3389/frph.2021.667413 36304017 PMC9580727

[56] Private sector delivery opportunities for the Dual Prevention Pill: Lessons from Family Planning for the introduction of Multi-purpose Prevention Technologies: AVAC; FP2030. (2022).

[57] Stoner MCD Rucinski KB Giovenco D Gill K Morton JF Bekker LG Trajectories of PrEP adherence among young women aged 16 to 25 in Cape Town, South Africa. AIDS Behav. (2021) 25(7):2046–53. 10.1007/s10461-020-03134-3 33389323 PMC8169554

[58] Mansoor LE Lewis L Naicker CL Harkoo I Dawood H Naidoo K Prospective study of oral pre-exposure prophylaxis initiation and adherence among young women in KwaZulu-Natal, South Africa. J Int AIDS Soc. (2022) 25(7):e25957. 10.1002/jia2.25957 35785472 PMC9251857

[59] Friedland BA Plagianos M Savel C Kallianes V Martinez C Begg L Women want choices: opinions from the share.learn.shape global internet survey about multipurpose prevention technology (MPT) products in development. AIDS Behav. (2023). 10.1007/s10461-022-03951-8. [Epub ahead of print] PMC10224828 36881183

[60] Ross J Stover J. Use of modern contraception increases when more methods become available: analysis of evidence from 1982 to 2009. Glob Health Sci Pract. (2013) 1(2):203–12. 10.9745/GHSP-D-13-00010 25276533 PMC4168565

[61] Yeh PT Kautsar H Kennedy CE Gaffield ME. Values and preferences for contraception: a global systematic review. Contraception. (2022) 111:3–21. 10.1016/j.contraception.2022.04.011 35525287 PMC9232836

[62] Ngure K. Choice and adherence to dapivirine ring or oral PrEP by young African women in REACH. Conference for retroviruses and opportunistic infections (CROI); virtual (2022).

[63] de Bekker-Grob EW Donkers B Bliemer MCJ Veldwijk J Swait JD. Can healthcare choice be predicted using stated preference data? Soc Sci Med. (2020) 246:112736. 10.1016/j.socscimed.2019.112736 31887626

[64] Sedgh G Hussain R. Reasons for contraceptive nonuse among women having unmet need for contraception in developing countries. Stud Fam Plann. (2014) 45(2):151–69. 10.1111/j.1728-4465.2014.00382.x 24931073

[65] Rothschild CW Richardson BA Guthrie BL Kithao P Omurwa T Mukabi J Contributions of side effects to contraceptive discontinuation and method switch among Kenyan women: a prospective cohort study. BJOG. (2022) 129(6):926–37. 10.1111/1471-0528.17032 34839583 PMC9035040

