# Supplementary material for: Synthesis of end-user research to inform future multipurpose prevention technologies in sub-Saharan Africa: a scoping review
Source: Front Reprod Health. 2023 May 31;5:1156864. doi: 10.3389/frph.2023.1156864 (PMC10264572; doi:10.3389/frph.2023.1156864)
Supplement: Supplementary file 1 [file Table1.docx]

Supplementary Material

Synthesis of End-User Research to Inform Future Multipurpose Prevention Technologies in sub-Saharan Africa: A Scoping Review

Nivedita L. Bhushan^*^, Kathleen Ridgeway, Ellen H. Luecke, Thesla Palanee-Phillips, Elizabeth T. Montgomery, Alexandra M. Minnis

*** Correspondence:** Nivedita L. Bhushan; [nbhushan@rti.org](mailto:nbhushan@rti.org)

Supplemental Table 1: Search Parameters for Peer Reviewed Literature

| **Domain** | **Reference Type** |
| --- | --- |
| Years | January 1, 2000 to November 30, 2022 |
| Language | English |
| Geography | Sub-Saharan Africa |
| Population | Women of Reproductive Age (15-24) |
| Key Words | - (MPT OR MPTs OR ((multipurpose OR multi-purpose) AND prevent* AND technolog*) OR dual (prevention OR protection) AND (delivery OR form OR forms OR type OR types OR attribute* OR preference* OR prefer* OR frequenc* OR duration OR side effect* OR adverse effect* OR menstrual OR menstruation OR fertility OR sexual* OR function* OR satisfaction OR satisfy* OR administration OR reversibility OR reversable OR efficacy OR safety OR value* OR cost OR costs OR economic* OR integrat* OR setting* OR messaging OR message* OR packaging OR brand* OR acceptability OR acceptable OR perspective*) - ("intravaginal ring*" OR "intra-vaginal ring*" OR "vaginal gel" OR "vaginal gels" OR "vaginal insert*" OR "vaginal film*" OR "rectal gel" OR "rectal gels" OR "implant" OR "implants" OR "microarray patch*" OR "micro-array patch*" OR "oral tablet*" OR "oral pill" OR "oral pills" OR injectable* OR "contraception" OR contraceptive* OR "birth control" OR "family planning") AND ("HIV" OR "human immunodeficiency virus") AND ("MPT" OR "MPTs" OR "multipurpose" OR "multi-purpose") - (partner OR partners OR family OR families OR community OR communities OR clinic OR clinics OR provider* OR physician* OR doctor* OR nurse* OR midwife* OR community health worker*) AND (MPT OR MPTs OR ((multipurpose OR multi-purpose) AND prevent* AND technolog*) OR dual (prevention OR protection) |
